# New susceptibility locus for obesity and dyslipidaemia on chromosome 3q22.3

**DOI:** 10.1186/1479-7364-7-15

**Published:** 2013-06-05

**Authors:** Maie Alshahid, Salma M Wakil, Mohammed Al-Najai, Nzioka P Muiya, Samar Elhawari, Daisy Gueco, Editha Andres, Samia Hagos, Nejat Mazhar, Brian F Meyer, Nduna Dzimiri

**Affiliations:** 1 King Faisal Heart Institute, MBC-16, King Faisal Specialist Hospital and Research Centre, Riyadh, 11211, Saudi Arabia; 2 Genetics Department, MBC-03, King Faisal Specialist Hospital and Research Centre, Riyadh, 11211, Saudi Arabia

**Keywords:** *MRAS* gene polymorphism, Coronary artery disease, Obesity, Hypercholesterolaemia, Dyslipidaemia, Hypertriglyceridaemia, Linkage, Haplotype

## Abstract

**Background:**

The muscle Ras (*MRAS*) gene resides on chromosome 3q22.3 and encodes a member of the membrane-associated Ras small GTPase proteins, which function as signal transducers in multiple processes including cell growth and differentiation. Its role in cardiovascular disease is not fully understood yet. In a preliminary study in heterozygous familial hypercholesterolaemia, we identified a locus linking the early onset of coronary artery disease (CAD) to chromosome 3q.22 and elected to sequence the *MRAS* gene using the MegaBACE DNA analysis system. In the present study, we investigated the association of seven single-nucleotide polymorphisms (SNPs) at this locus with CAD and its dyslipidaemia-related risk traits in 4,650 Saudi angiographed individuals using TaqMan assays by the Applied Biosystems real-time Prism 7900HT Sequence Detection System.

**Results:**

Among the studied SNPs, rs6782181 (*p* = 0.017) and rs9818870T (*p* = 0.009) were associated with CAD following adjustment for sex, age and other confounding risk factors. The rs6782181_GG also conferred risk for obesity (1,764 cases vs. 2,586 controls) [1.16(1.03–1.30); *p* = 0.017], hypercholesterolaemia (1,686 vs. 2,744) [1.23(1.02–1.47); *p* = 0.019], hypertriglyceridaemia (1,155 vs. 3,496) [1.29(1.01–1.45); *p* = 0.043] and low high-density lipoprotein-cholesterol (lHDL-chol) levels (1,935 vs. 2,401) [1.15(1.02–1.30); *p* = 0.023] after adjustment. Additionally, rs253662_(CT+TT) [1.16(1.01–1.32); *p* = 0.030] was associated with lHDL-chol levels. Interestingly, rs253662 (*p* = 0.014) and rs6782181 (*p* = 0.019) were protective against acquiring high low-density lipoprotein-cholesterol (hLDL-chol) levels (*p* = 0.014), while rs1720819 showed similar effects against CAD (*p* < 0.0001). More importantly, a 7-mer haplotype, ACCTGAC (*χ*^2^ = 7.66; *p* = 0.0056), constructed from the studied SNPs, its 6-mer derivative CCTGAC (*χ*^2^ = 6.90; *p* = 0.0086) and several other shorter derivatives conferred risk for obesity. hLDL-chol was weakly linked to CTAA (*χ*^2^ = 3.79; *p* = 0.052) and CCT (*χ*^2^ = 4.32; *p* = 0.038), while several other haplotypes were protective against both obesity and hLDL-chol level.

**Conclusion:**

Our results demonstrate that the genomic locus for the *MRAS* gene confers risk for CAD, obesity and dyslipidaemia and point to the possible involvement of other genes or regulatory elements at this locus, rather than changes in the M-Ras protein function, in these events.

## Background

The *MRAS* gene encodes a member of the membrane-associated family of Ras small GTPase proteins [1], which function as signal transducers in multiple processes including cell growth and differentiation [2-6]. With the growing number of effectors being continually described for this GTP-binding protein superfamily [7,8], it is becoming increasingly evident that their signal transduction leads to the generation of a multitude of cellular signals. Among others, these proteins play a role in the tumour necrosis factor-alpha and MAP kinase signalling pathways [9-12]. Hence, the dysregulation of the Ras signalling has been associated with many types of cancers [13-17]. Notably, the M-Ras protein is ubiquitously expressed but very highly so in the cardiovascular system, particularly in the heart itself [18], intuitively pointing to a possibly pivotal role in cardiovascular function. However, its role in cardiovascular disease is not fully understood yet. A recent study by Erdmann et al. [19] revealed a region on 3q22.3, which encompasses the *MRAS* gene, as a risk factor for coronary artery disease (CAD), while another study by Ellis et al. [20] proposed that the rs9818870 at this locus is a predictor of cardiovascular risk in individuals free of overt heart disease. Besides, a previous study has shown that M-Ras is engaged in tumour necrosis factor-alpha-stimulated lymphocyte function-associated antigen 1 activation in splenocytes [10], thus suggesting a role in adhesion signalling, which is an important aspect of atherosclerotic pathways [21].

Currently, there is hardly any tangible data available in the literature pertaining to the possible role of the *MRAS* gene polymorphism in cardiovascular risk traits for CAD, such as obesity, hypertension (HTN), type 2 diabetes mellitus (T2DM) or dyslipidaemic disorders. In a linkage study on the early onset of CAD in heterozygous familial hypercholesterolaemia (HFH), we identified a locus on chromosome (chr) 3q.22 that was linked to both disorders (Dzimiri et al., unpublished data). Considering the fact that this locus has also been implicated in CAD, our present study addressed the question as to whether it might signify a link between dyslipidaemia-related disorders and atherosclerosis onset in general, in a homogenous cohort of 4,650 Saudi individuals harbouring CAD and its lipid-metabolic risk traits.

## Results

**Figure 1:**
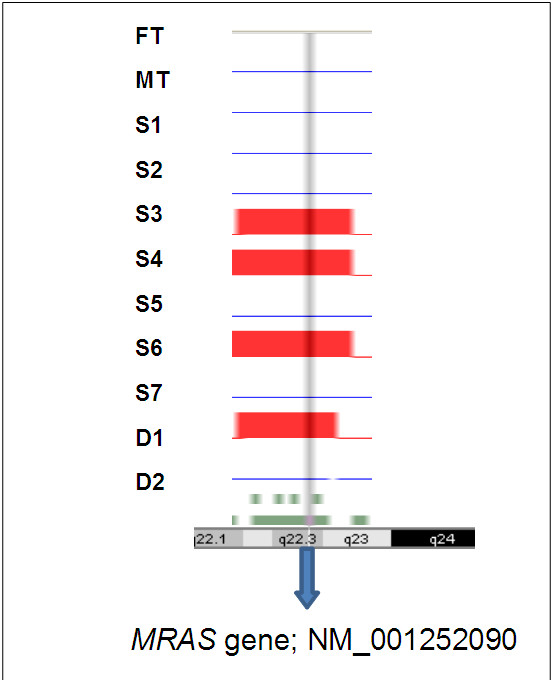
**Homozygosity mapping for early onset of coronary artery disease in heterozygous familial hypercholesterolaemia.** Affymetrix GT console mapping indicating the position of homozygosity for the four affected offsprings: S3, S4, S6 and D1. FT, father; MT, mother; S1–7, sons 1–7; D1 and 2, daughters 1 and 2.

**Figure 2:**
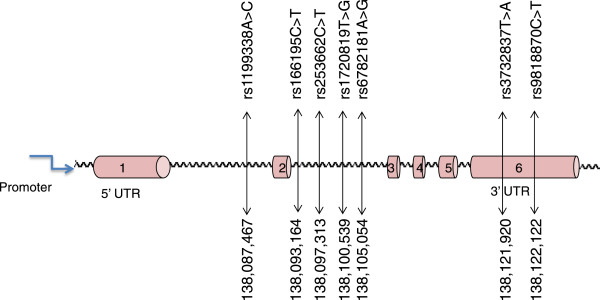
**Schematic diagram of the MRAS (not to scale).** The figure shows studied SNPs sequentially arranged according to their chromosomal position (based on transcript NM_001252090). These SNPs were detected with a prevalence of >0.1 in the general population.

**Figure 3:**
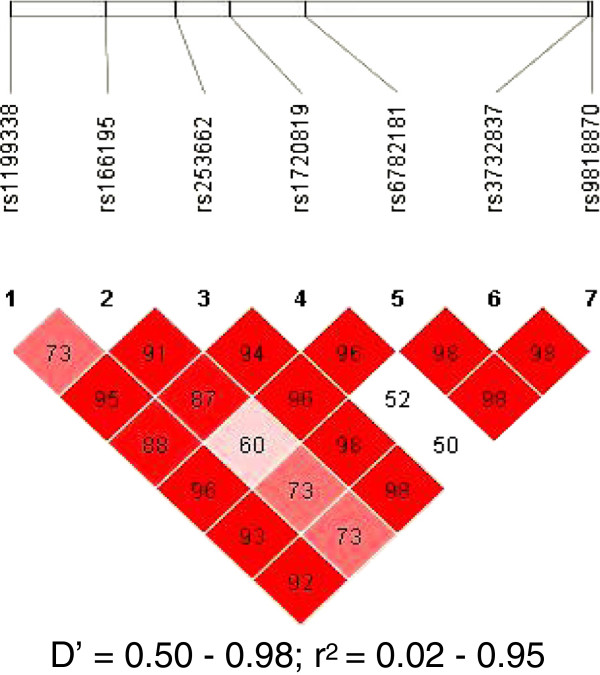
**Linkage disequilibrium of the seven studied SNPs.** The figure displays the coefficient of the linkage disequilibrium (*D*’) and the regression coefficient of the linkage disequilibrium (*r*^2^) for the studied SNPs.

The initial linkage study using the Affymetrix Gene Chip 250 sty1 mapping array revealed several genomic loci, including one on chr 3q.22, as a potential risk locus for both the early onset of CAD and familial hypercholesterolaemia in a family of 11 members harbouring HFH (Figure 1). We then selected the *MRAS*, which resides at this locus, as the target gene for further studies on its role as a risk for both CAD and dyslipidaemic disorders. This was followed by sequencing the gene in the family members and 200 individuals from the general population in order to identify informative single-nucleotide polymorphisms (SNPs) of interest. Our results indicated that the *MRAS* gene is extensively polymorphic in the Saudi population, yielding several SNPs in both the coding and non-coding areas of the gene. Particularly noteworthy was the finding of a large number of intronic SNPs as well as those residing in the 3 prime untranslated region (3′-UTR) of the gene. This stimulated an interest for us to examine the likelihood that these regions may constitute significant risk loci for complex diseases, such as CAD. Accordingly, we then selected seven SNPs showing minor allele frequencies of >0.1 in these individuals, as potentially informative for their possible association with disease. Apart from their frequencies in the above groups, the selection of these SNPs was also partly based on available information on their impact on disease to allow comparison with other data in the literature. Selected SNPs were rs1199338_AC (1), rs166195_CT (2), rs253662_CT (3), rs1720819_TG (4), rs6782181_AG (5), rs3732837_TA (6) and rs9818870_CT (7), numbered sequentially according to their chromosomal positional arrangement (Figure 2). The linkage disequilibrium for the SNPs is given in Figure 3.

**Figure 4:**
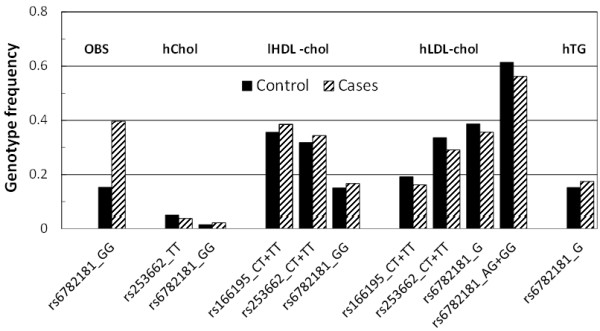
Association of MRAS genotypes with disease traits.

The association analysis was performed in a total of 4,650 angiographed individuals, of which 2,429 had CAD and 2,221 served as CAD-free controls. Among the studied SNPs, rs6782181 (*p* = 0.017) and rs9818870T (*p* = 0.009) were associated with CAD following adjustment for sex, age and the confounding effects of other risk factors (Table 1; see also Additional file 1). Notably, rs6782181GG also conferred risk for obesity (1,764 cases vs. 2,586 controls) [1.16(1.03–1.30); *p* = 0.017], hypercholesterolaemia (hChol; 1,686 vs. 2,744) [1.23(1.02–1.47); *p* = 0.019], hypertriglyceridaemia (hTG; 1,166 vs. 3,168) [1.29(1.01–1.45); *p* = 0.043] and low high-density lipoprotein-cholesterol (lHDL-chol) levels (1,935 vs. 2,401) [1.15(1.02–1.30); *p* = 0.023] following adjustment for the possible confounding effects of other risk factors (Table 2; Figure 4). Additionally, rs253662 (CT+TT) [1.16(1.01–1.32); *p* = 0.030] was also implicated in the harbouring of lHDL-chol levels. One other SNP, rs166195, lost its association with lHDL-chol following the adjustment for confounders. Interestingly, two of the SNPs, rs253662 (*p* = 0.014) and rs6782181 (*p* = 0.019), were protective against acquiring hLDL-chol, whereby the former showed similar but weaker properties towards hypercholesterolaemia. One other variant, rs1720819, was protective against CAD (*p* < 0.0001). Furthermore, none of the variants showed any delineable relationship with hypertension or type 2 diabetes mellitus.

**Table 1:** **Association of *MRAS *variants with coronary artery disease**

**Model**	**Unstandardized coefficient**		**Standardized coefficient**	***t***	***p *value**	**95.0% C.I. for beta**	
	**Beta**	**S.E.**	**Beta**			**Lower bound**	**Upper bound**
Sex	−0.107	0.009	−0.102	−12.05	0.000***	−0.125	−0.09
Age	0.003	0.000	0.081	8.53	0.000***	0.002	0.003
FH	−0.004	0.01	−0.003	−0.401	0.689	−0.024	0.016
T2DM	0.082	0.009	0.082	9.034	0.000***	0.064	0.1
MI	0.586	0.01	0.56	59.874	0.000***	0.567	0.605
HTN	0.03	0.01	0.026	2.907	0.004***	0.01	0.05
OBS	−0.018	0.008	−0.018	−2.177	0.03*	−0.035	−0.002
rs119338_C	0.002	0.023	0.002	0.106	0.915	−0.043	0.048
rs166195_C	0.053	0.049	0.045	1.076	0.282	−0.044	0.15
rs166195_T	0.038	0.048	0.033	0.785	0.433	−0.057	0.133
rs253662_T	−0.012	0.019	−0.009	−0.622	0.534	−0.049	0.026
rs1720819_T	−0.182	0.052	−0.119	−3.505	0.000***	−0.284	−0.08
rs1720819_G	−0.164	0.055	−0.104	−2.996	0.003**	−0.272	−0.057
rs6782181_AA	0.073	0.031	0.071	2.379	0.017*	0.013	0.133
rs6782181_T	0.057	0.031	0.055	1.816	0.069	−0.005	0.118
rs3732837_AA	0.013	0.022	0.009	0.58	0.562	−0.03	0.056
rs3732837_T	−0.027	0.043	−0.017	−0.643	0.52	−0.111	0.056
rs9818870_C	0.097	0.04	0.063	2.416	0.016*	0.018	0.176
rs9818870_T	0.14	0.054	0.087	2.612	0.009**	0.035	0.246

The table shows the significant association of the *MRAS* gene variants with CAD following Bonferroni correction. Beta, odds ratio coefficient; *FH* family history of CAD, *T2DM* type 2 diabetes mellitus, *MI* myocardial infarction, *HTN* hypertension, *OBS* obesity, *C.I* confidence interval, *S.E.* standard error; *t*, standard *t* test. *p < 0.05; **p < 0.01; ***p < 0.005.

**Table 2:** **Association of *MRAS *gene with obesity and dyslipidaemia**

**Variant**	**Controls**	**Cases**	**Univariate analysis**		**Multivariate analysis**	
			**Exp( *B *) (95% C.I.)**	***p *value**	**Exp( *B *) (95% C.I.)**	***p *value**
Obesity						
rs6782181_GG	0.153	0.397	1.15(1.02–1.29)	0.023*	1.16(1.03–1.30)	0.017*
Hypercholesterolaemia						
rs253662_TT	0.051	0.038	0.73(0.59–0.90)	0.041*	0.74(0.53–1.01)	0.054
rs6782181_GG	0.015	0.022	1.47(1.07–2.03)	0.018*	1.23(1.02–1.47)	0.019*
Low HDL levels						
rs166195_CT+TT	0.356	0.385	1.14(1.00–1.29)	0.047*	1.00(0.99–1.00)	0.961
rs253662_CT+TT	0.318	0.344	1.13(0.99–1.29)	0.064	1.16(1.01–1.32)	0.030*
rs6782181_GG	0.151	0.167	1.13(1.00–1.27)	0.045*	1.15(1.02–1.30)	0.023*
High LDL-cholesterol						
rs253662_T	0.192	0.162	0.82(0.70–0.96)	0.013*	0.82(0.69–0.96)	0.014*
rs253662_CT+TT	0.336	0.291	0.82(0.67–0.98)	0.028*	0.81(0.68–0.98)	0.030*
rs6782181_G	0.387	0.356	0.88(0.77–0.99)	0.038*	0.88(0.77–0.99)	0.040*
rs6782181_AG+GG	0.614	0.564	0.82(0.68–0.97	0.019*	0.81(0.68–0.97)	0.019*
Hypertriglyceridaemia						
rs6782181_G	0.152	0.175	1.19(1.00–1.42)	0.067	1.29(1.01–1.45)	0.043*

The table shows the *MRAS* variants associated with the disease traits. B, odds ratio; C.I., confidence interval. *p < 0.05.

We were also interested in evaluating whether any haplotype was identifiable in relationship to these risk traits. We employed the most common 7-mer haplotype, ACCTAAC (frequency = 0.558), constructed from the studied SNPs as a baseline for comparing their relationships with the disease traits. Interestingly, a 7-mer haplotype, ACCTGAC (*χ*^2^ = 7.66; *p* = 0.0056), its 6-mer derivative CCTGAC (*χ*^2^ = 6.90; *p* = 0.0086) and other shorter derivatives conferred risk for obesity. Another 7-mer haplotype, ACCTAAC (*χ*^2^ = 4.63; *p* = 0.031), and its derivatives were protective against obesity (Table 3; Additional file 2). Besides, no notable causative association was observed between the haplotypes and changes in total cholesterol level. Notably, apart from a 3-mer CCT (*χ*^2^ = 4.32; *p* = 0.038) that was positively associated with hLDL-chol levels, the only other haplotypes linked, but weakly so, to this trait were the 4-mer TAAC (*p* = 0.057) and its 3-mer derivative AAC (*p* = 0.052), both of which were actually protective towards obesity (*χ*^2^ = 4.40; *p* = 0.036). Instead, two 7-mer haplotypes, ATTTGAC (*χ*^2^ = 4.39; *p* = 0.036) and CCCGGTT (*χ*^2^ = 4.31; *p* = 0.038), showed protective properties towards acquiring hLDL-chol levels.

**Table 3:** **Association of *MRAS *haplotypes with obesity and high low-density lipoprotein levels**

**Block**	**Haplotype**	**Pooled**	**Cases**	**Control**	***χ***^**2**^	***p *value**
Obesity						
1–7	ACCTAAC	0.558	0.541	0.565	4.63	0.031
	ACCTGAC	0.035	0.042	0.031	7.66	0.0056*
1–6	ACCTAA	0.558	0.541	0.564	4.60	0.032
	ACCTGA	0.036	0.044	0.032	8.28	0.004**
2–7	CCTAAC	0.56	0.543	0.567	4.61	0.032
	CCTGAC	0.035	0.042	0.032	6.90	0.0086*
3–7	CTAAC	0.614	0.597	0.618	3.74	0.053
	CTGAC	0.04	0.047	0.036	7.15	0.0075*
1–5	ACCTA	0.558	0.541	0.564	4.36	0.039
	ACCTG	0.037	0.045	0.033	9.22	0.0024**
4–7	TAAC	0.618	0.601	0.623	4.40	0.036
High LDL-cholesterol						
1–7	ATTTGAC	0.172	0.153	0.177	4.39	0.036
	CCCGGTT	0.058	0.045	0.060	4.31	0.038
2–7	TTTGAC	0.173	0.152	0.179	5.11	0.024
	CCGGTT	0.062	0.046	0.064	6.27	0.012
1–6	ATTTGA	0.172	0.152	0.177	4.54	0.033
1–5	ATTTG	0.172	0.152	0.176	4.49	0.034
2–6	TTTGA	0.173	0.151	0.178	5.45	0.019
4–7	TAAC	0.618	0.640	0.612	3.61	0.057
	GGTT	0.061	0.048	0.064	4.81	0.028
1–4	ATTT	0.174	0.153	0.179	4.92	0.027
3–6	CTAA	0.613	0.635	0.606	3.79	0.052
	TTGA	0.181	0.158	0.187	6.02	0.014
2–5	TTTG	0.174	0.152	0.179	5.23	0.022
1–3	ATT	0.175	0.154	0.180	5.01	0.025
5–7	AAC	0.620	0.642	0.613	3.78	0.052
2–4	CCT	0.638	0.663	0.632	4.32	0.038

The table shows selected haplotypes constructed from combinations of the studied variants. The most frequent 7-mer haplotype, ACCTAAC (0.558), was employed as the baseline haplotype to determine the relative effects of the associated haplotypes. The minor allele frequency and control and case haplotype frequencies are proportions. Number 1 denotes rs1199338_AC, 2 is rs166195_CT, 3 is rs253662_CT, 4 is rs1720819_TG, 5 is rs6782181_AG, 6 is rs3732837_AT and 7 is rs9818870_CT, arranged sequentially by their chromosomal positions, and blocks represent the variant constituting the respective haplotypes. *p < 0.01; **p < 0.005 by the *χ*^2^ test.

## Discussion

The present study evaluated the role of gene variants residing at the genomic locus of the *MRAS* gene in CAD and its metabolic risk factors. Our results indicate that at least two of the studied SNPs were associated with CAD. The finding of an association with CAD in the present study is in agreement with the association of a cluster of four SNPs consisting of rs1199338, rs2347252, rs3732837 and rs9818870 in this chromosomal region with CAD in the study of Erdmann et al. [19]. More importantly, our study also revealed that, apart from CAD, this locus is linked to its important risk traits, obesity, and the lipidaemic disorders, hyperlipidaemia, hypertriglyceridaemia and the harbouring of low HDL-cholesterol levels. Notably, one predisposing SNP, rs6782181, was common to CAD, obesity and the dyslipidaemia traits, both independently and following correction for confounding factors. Thus, our data not only describe a relationship for this SNP with three important dyslipidaemia-related risk traits, but also suggest a possible genomic link for these cardiovascular disease traits at this locus. Besides, lHDL-chol levels were further associated with the dominant inheritance mode for rs253662 and weakly linked to that for the rs166195. Therefore, put together, our results point to the genomic locus for the *MRAS* gene as conferring risk for CAD and its important metabolic risk traits.

Interestingly, the association of rs253662 with the harbouring of low HDL-cholesterol levels is in direct contrast to its protective effects on the acquisition of high LDL-cholesterol and similar but weaker properties towards elevated total cholesterol levels. Currently, apart from a study implicating the rs9818870 as a predictor of cardiovascular risk in individuals free of overt heart disease, there is hardly any literature describing the relationship between the *MRAS* gene or this genomic locus and lipid metabolic disorders. Thus, to our knowledge, this is the first report implicating this region in obesity and dyslipidaemia.

We further evaluated the possibility that haplotypes might be more informative than the individual SNPs in discerning the impact of the gene on disease. It was remarkable that a haplotype constructed from these SNPs and several of its shorter derivatives conferred risk for obesity, displaying a much higher level of significance for the relationships than the individual variants. Even more unique was the observation that the only trait unequivocally associated with these haplotypes was obesity. If anything, for the other disease traits, the large majority of the associations were actually protective against acquiring high LDL-chol, in line with the observed protective actions of the individual SNPs. These observations can be conceived as confirming the importance of this genomic region as a specific risk for obesity. The weak association of haplotypes with the dyslipidaemic traits may indicate that the influence of the gene on dyslipidaemia-related events may be driven primarily through the interaction of the gene with obesity.

The interesting question is whether or not this locus might provide a mechanistic link between dyslipidaemia and the manifestation of CAD. A number of observations seem to suggest a direct link of the mechanisms involved in these events at the genomic level to some function of this locus. Particularly noteworthy is the fact that the studied variants are either intronic or reside in the 3′-UTR relative to the *MRAS* gene sequence. This implies that the effects of the studied variants are not likely to be directly related to functional changes of M-Ras protein *per se*. Thus, while no speculations can be made of the mechanisms involved in these interactions, based on the current findings alone, it can nonetheless be inferred that other yet unidentified genes/entities at the chromosome 3q.22 locus may ultimately be responsible for the observed manifestations and might offer a link between dyslipidaemia and pathways leading to atherosclerosis. Such mechanisms, particularly those related to SNPs in the 3′-UTR may involve some gene regulatory entities embedded in the function of this genomic region. Genetic hitchhiking might also explain the association of particularly the intragenic non-coding SNPs, as a result of the variants being linked with a causative mutation on the M-Ras through positive selection at this locus [22-24] or some spatial selection pressures [25-29]. However, further speculation on this possibility is beyond the scope of the present work. Hence, further studies are needed to look into these possibilities.

## Conclusions

In conclusion, our study suggests that the genomic locus for the *MRAS* gene confers risk for CAD, obesity and dyslipidaemia. However, our findings point to the involvement of other genes or factors, rather than changes in the M-Ras protein function, as the possible underlying cause for the observed events.

## Methods

### Study population

The initial linkage study was performed in a family of 11 individuals with HFH, in which the primary proband underwent triple bypass surgery at the age of 14 years. Following the identification of chromosome 3q.22 as a potential locus for this disorder as well as the early onset of CAD, we elected to sequence the *MRAS* gene in the family and 200 other individuals from the general population. We then embarked on a case-control study in a total of 4,650 individuals consisting of 2,429 CAD patients (1,860 males and 569 females, mean age 55.6 ± 0.4 years) with angiographically determined narrowing of the coronary vessels by at least 50% and 2,221 angiographed controls (1,189 males and 1,032 females, mean age 53.8 ± 0.5 years). The controls for CAD were a group of individuals undergoing surgery for heart valvular diseases, and those who may have reported with chest pain, but were established to have no significant coronary stenosis by angiography. Among these, 3,060 individuals had established MI. The primary patient subset of interest in this population comprised 1,764 obese individuals with a body mass index of ≥30.0 kg/m^2^ (Table 4). Furthermore, 3,541 individuals had primary (essential) HTN, defined as ≥140 mmHg systolic blood pressure or ≥90 mmHg diastolic pressure based on The Sixth Report of the Joint National Committee on Prevention, Detection, Evaluation, and Treatment of High Blood Pressure (JNC VI) criteria [30]. Accordingly, essential, primary or idiopathic hypertension is defined as high blood pressure in which secondary causes such as renovascular disease, renal failure, pheochromocytoma, aldosteronism or other causes of secondary hypertension or Mendelian forms (monogenic) are not present [30]. The third subset of interest was composed of 2,560 individuals with T2DM (formerly known as non-insulin-dependent diabetes mellitus or adult-onset diabetes). The National Diabetes Data Group of the USA and the Second World Health Organization Expert Committee on Diabetes Mellitus [31] defined type 2 diabetes mellitus as a metabolic disorder that is characterized by high blood glucose (generally defined as a fasting glucose level >126 mg/dl) in the context of insulin resistance and relative insulin deficiency. Among these subsets of patients, some individuals harboured a combination of two or possibly three of the cardiovascular risk traits. The overall exclusion criteria for the disease cases were major cardiac rhythm disturbances, incapacitating or life-threatening illness, major psychiatric illness or substance abuse, history of cerebral vascular disease, neurological disorder and the administration of psychotropic medication. Exclusion criteria for the controls were, among others, diseases such as cancer, autoimmune disease or any other disorders likely to interfere with the variables under investigation. All participants signed an informed consent, and the study was performed in accordance with the regulations laid down by the King Faisal Specialist Hospital and Research Centre Ethics Committee in compliance with the Helsinki Declaration [32].

**Table 4:** Demographics and clinical data of the studied patients

	**Controls**			**Cases**		
	**All**	**Male**	**Female**	**All**	**Male**	**Female**
Demographics						
OBS	2,586	1,886(0.73)	700(0.27)	1,764	967(0.55)	797(0.45)
Age^a^	53.8 ± 0.5	56.4 ± 0.3	51.1 ± 0.7	55.6 ± 0.4	55.5 ± 0.4	55.7 ± 0.4
BMI^a^	25.3 ± 0.1	25.3 ± 0.1	25.2 ± 0.1	34.9 ± 0.2	33.8 ± 0.1	35.9 ± 0.2
CAD	2,221	1,189(0.54)	1,032(0.46)	2,429	1,860(0.66)	569(0.34)
MI	1,590	787(0.49)	803(0.51)	3,060	2,262(0.74)	798(0.26)
FH	3,730	2,449(0.66)	1,283(0.34)	920	602(0.65)	318(0.35)
T2DM	2,090	1,336(0.64)	754(0.36)	2,560	1,713(0.55)	847(0.45)
HTN	1,109	735(0.66)	374(0.34)	3,541	2,314(0.65)	1,227(0.35)
lHDLC	2,516	1,487(0.59)	1,029(0.41)	1,935	1,487(0.77)	448(0.23)
hLDLC	3,696	2,449(0.75)	1,029(0.25)	860	634(0.74)	226(0.26)
hChol	2,744	1,801(0.66)	943(0.34)	1,686	1,117(0.66)	569(0.34)
hTG	3,168	2,028(0.64)	1,140(0.36)	1,166	829(0.71)	337(0.29)
Smokers	2,791	1,314(0.47)	1,477(0.53)	1,771	1,694(0.96)	77(0.04)
Clinical characteristics (relative to coronary artery disease)						
Age	50.6 ± 0.4	51.2 ± 0.5	49.8 ± 0.5	60.3 ± 0.2	59.8 ± 0.3	61.8 ± 0.54
BMI	29.0 ± 0.2	27.97 ± 0.2	30.3 ± 0.3	28.9 ± 0.1	28.3 ± 0.1	31.0 ± 0.3
Total chol	4.51 ± 0.02	4.42 ± 0.03	4.62 ± 0.03	4.48 ± 0.02	4.43 ± 0.03	4.66 ± 0.05
HDL-chol	1.26 ± 0.01	1.18 ± 0.03	1.33 ± 0.01	1.15 ± 0.01	1.15 ± 0.01	1.25 ± 0.02
LDL-chol	2.76 ± 0.02	2.73 ± 0.03	2.80 ± 0.03	2.71 ± 0.02	2.68 ± 0.02	2.84 ± 0.06
TG	1.52 ± 0.02	1.60 ± 0.03	1.44 ± 0.03	1.78 ± 0.02	1.78 ± 0.03	1.78 ± 0.05
FG	6.87 ± 0.16	6.80 ± 0.23	6.92 ± 0.22	9.45 ± 0.31	9.27 ± 0.37	9.88 ± 0.56
BP	120/83	119/81	121/82	128/84	130/85	127/83

The numbers in brackets give the fraction of the total (all) values of the group. *BMI* body mass index, *CAD* coronary artery disease, *FH* family history of CAD, *MI* myocardial infarction, *FG* fasting glucose, T2DM type 2 diabetes mellitus, lHDLC low high-density lipoprotein-cholesterol, hLDLC high low-density lipoprotein-cholesterol, hTG hypertriglyceridaemia, hChol hypercholesterolaemia, HTN hypertension. ^a^Values relative to obesity as the case group.

### Linkage analysis and screening for mutations

Five millilitres of peripheral blood was sampled in EDTA tubes from each of the study individuals after obtaining their written consent, and genomic DNA was extracted from leukocytes by the standard salt method using Gentra PUREGENE DNA isolation kit (Qiagen Sciences, Germantown, MD, USA). For the genome-wide scanning with the Affymetrix Gene Chip 250 sty1 mapping array (Affymetrix Inc., Santa Clara, CA, USA), 250 ng of genomic DNA was digested with the restriction endonuclease *Sty*I, mixed with Sty1 adaptors and ligated with T4 DNA ligase. The mixture was added to four separate polymerase chain reactions (PCRs), amplified, pooled and purified to remove the unincorporated dNTPs. The PCR product was then fragmented, biotinylated, hybridized to the 250 sty1 array for 18 h, washed, stained and scanned as recommended by the manufacturer. SNP genotypes, linear chromosomal locations and marker ordering were accomplished using the Affymetrix Genotyping Console (GC) Software version 3.02 (Affymetrix, Inc., Santa Clara, CA, USA). Extended blocks or regions of homozygosity (ROHs) were identified using the GC software. Conventionally, ROHs are defined as stretches of consecutive alleles in affected individuals and heterozygous or homozygous for the other allele in unaffected members of the same family. For the linkage analysis, multipoint parametric linkage analysis was performed using the GeneHunter Easy Linkage Analysis Software 4.0 module for estimating the LOD scores. The disease was assumed to be an autosomal dominant trait with 100% penetrance. Copy Number Analyzer for GeneChip® Ver. 3.0 (Affymetrix, Inc., Santa Clara, CA, USA) was employed to check the shared chromosomal regions of homozygosity.

### Sequencing of the *MRAS* gene

The sequencing of the *MRAS* gene was accomplished using the MegaBACE DNA analysis system (Amersham Biosciences, Sunnyvale, CA, USA). Briefly, the DNA was subjected to PCR by standard methods described elsewhere. Five microlitres of PCR product was then treated with 2 μl of ExoSAP-IT (USB Corporation, Cleveland, OH, USA) at 37°C for 30 min to allow the hydrolytic removal of excess primers by exonuclease 1 and shrimp alkaline phosphatase. The enzymes were inactivated at 80°C for 15 min, and the sequencing reaction was initiated by mixing 2 μl of DNA, 1 μl of 5 μmol primer, 8 μl of DYEnamic ET Dye Terminator (Amersham Biosciences, Amersham, Buckinghamshire, UK) and 9 μl of distilled water. The mixture was thermally cycled 40× at 95°C for 20 s, 50°C for 15 s and 60°C for 1 min. Unincorporated dye-labelled terminators were removed by gel filtration through the DyeEx 96 plate (Qiagen, GmbH, Hilden, Germany). The eluent was vacuum-dried and dissolved in 10 μl of loading solution (GE Healthcare UK Ltd, Little Chalfont, Buckinghamshire, UK) for sequencing. Data were analyzed for SNPs using the Lasergene software (DNASTAR, Inc., Madison, WI, USA).

### Association experiments

Once the SNPs of interest were identified, genotyping was achieved by TaqMan chemistry using the Applied Biosystems real-time Prism 7900HT Sequence Detection System (ABI Inc., Foster City, CA, USA). Primers and the TaqMan fluorogenic probes bearing a suitable reporter dye on the 5′-end and a quencher dye on the 3′-end were designed using the Primer Express Software V2.0 (ABI Inc., Foster City, CA, USA) and procured from Applied Biosystems (ABI, Warrington, UK). One probe (for allele 1) was labelled with VIC dye and the other (for allele 2) with FAM dye at the 5′-end, and serial dilutions were run to determine the optimal working concentration. For each reaction, a 25-μl reaction was prepared by mixing 5 μl of 50 ng DNA, 12.5 μl of 2× Universal mix (Eurogentec, Liege Science Park, Seraing, Belgium), 1.25 μl of 20× probe assay mix and 6.25 μl of DNase-free distilled water. Three no-template controls were included in each plate for the normalization of emission signal. The thermal profile for amplification for the first cycle occurred at 50°C for 2 min and 95°C for 10 min, followed by 40 cycles of 94°C for 15 s and 60°C for 30 s. The plates were then scanned for FRET signal using the 7900HT Sequence Detection System and data analyzed using the SDS 2.0 software (ABI Inc., Foster City, CA, USA).

### Statistical analysis

Comparison of genotypes and alleles between different groups for continuous dependent variables was achieved by analysis of variance or Student's *t* test as appropriate. Categorical variables were analyzed by chi-square test. Univariate and multiple logistic regression analyses were used to compute odds ratios and their 95% confidence intervals as well as to determine the confounding effects of the different cardiovascular risk traits on the individual respective relationships. The haplo.stats package [33] in the R Statistical Computing software [34] was used to perform haplotype-based association analysis. Odds ratios for haplotypes were calculated using as reference the most frequent 7-mer haplotype, ACCTAAC (frequency = 0.558), and the Haplotype Score statistic for the association of a haplotype with the binary trait was calculated as in Schaid et al. [35] and Lake et al. [36]. The significance of association was determined between haplotypes and the case-control status - a binomial trait denoting whether or not a patient had the disease. All other statistical analyses were performed using the SPSS version 20 software (SPSS Inc., Chicago, IL, USA), and data are expressed as mean ± SEM. Associations with a two-tailed *p* value <0.05 were considered statistically significant.

## Abbreviations

3′-UTR: 3 prime untranslated region; BP: Blood pressure; CAD: Coronary artery disease; FG: Fasting glucose; FH: Family history of CAD; hChol: Hypercholesterolaemia; HDLC: High-density lipoprotein-cholesterol; hTG: Hypertriglyceridaemia; HTN: Hypertension; LDLC: Low-density lipoprotein-cholesterol; MI: Myocardial infarction; MRAS: Membrane-associated muscle Ras small GTPase gene; OBS: Obesity; SNP: Single-nucleotide polymorphism; T2DM: Type 2 diabetes mellitus.

## Competing interests

The authors declare that they have no competing interests.

## Authors’ contributions

MA supervised the recruitment of the patients and compliance with institutional ethical procedures. SMW performed the gene mapping analysis. MA-N was responsible for overall running the TaqMan assays. NPM was involved in running the Affymetrix assays, designing the probes, screening for gene mutations as well as participating in the write up of the manuscript. SE performed part of the sequencing experiments. SH ran the Affymetrix assays. DG contributed to running the TaqMan assays. EA performed the DNA isolation and maintained the patient database. NM was responsible for clinical patient data and material acquisition. BFM contributed to the write up of the manuscript. ND is the principal investigator, with the overall responsibility of the project and the preparation of the manuscript. All authors read and approved the final manuscript.

## Supplementary Material

Additional file 1 **Statistical analysis for the association of *MRAS* variants with disease.** The file contains the analyses of the *MRAS* gene with coronary artery disease and obesity and the univariate and multivariate analyses for the *MRAS* variants displaying a significant association with the disease traits. Click here for file

Additional file 2 **Analysis of the association of *MRAS* haplotypes with disease.** The file contains a table showing the haplotypes constructed from combinations of the studied variants with respect to obesity. Click here for file
