# Supplementary material for: New susceptibility locus for obesity and dyslipidaemia on chromosome 3q22.3
Source: Hum Genomics. 2013 Jun 5;7(1):15. doi: 10.1186/1479-7364-7-15 (PMC3681549; doi:10.1186/1479-7364-7-15)
Supplement: Additional file 1 — Statistical analysis for the association of MRAS variants with disease. The file contains the analyses of the MRAS gene with coronary artery disease and obesity and the univariate and multivariate analyses for the MRAS variants displaying a significant association with the disease traits. [file 1479-7364-7-15-S1.docx]

***Suppl data***

1. ***Analysis for MRAS with coronary artery disease***

| Suppl Table 1: *Relationship of CAD with MRAS and different risk factors in the studied population* | | | | | | | |
| --- | --- | --- | --- | --- | --- | --- | --- |
| Model | Unstandardized Coefficients | | Standardized Coefficients | t | P-Value | 95.0% Confidence Interval for B | |
|  | B | Std. Error | Beta |  |  | Lower Bound | Upper Bound |
| Sex | -0.107 | 0.009 | -0.102 | -12.05 | .000 | -0.125 | -0.09 |
| Age | 0.003 | .000 | 0.081 | 8.53 | .000 | 0.002 | 0.003 |
| FH | -0.004 | 0.01 | -0.003 | -0.401 | 0.689 | -0.024 | 0.016 |
| DM | 0.082 | 0.009 | 0.082 | 9.034 | .000 | 0.064 | 0.1 |
| MI | 0.586 | 0.01 | 0.56 | 59.874 | .000 | 0.567 | 0.605 |
| HTN | 0.03 | 0.01 | 0.026 | 2.907 | 0.004 | 0.01 | 0.05 |
| OBS | -0.018 | 0.008 | -0.018 | -2.177 | 0.03 | -0.035 | -0.002 |
| rs119338C | 0.002 | 0.023 | 0.002 | 0.106 | 0.915 | -0.043 | 0.048 |
| rs166195C | 0.053 | 0.049 | 0.045 | 1.076 | 0.282 | -0.044 | 0.15 |
| rs166195T | 0.038 | 0.048 | 0.033 | 0.785 | 0.433 | -0.057 | 0.133 |
| rs253662T | -0.012 | 0.019 | -0.009 | -0.622 | 0.534 | -0.049 | 0.026 |
| rs1720819T | -0.182 | 0.052 | -0.119 | -3.505 | .000 | -0.284 | -0.08 |
| rs1720819G | -0.164 | 0.055 | -0.104 | -2.996 | 0.003 | -0.272 | -0.057 |
| rs6782181AA | 0.073 | 0.031 | 0.071 | 2.379 | 0.017 | 0.013 | 0.133 |
| rs6782181T | 0.057 | 0.031 | 0.055 | 1.816 | 0.069 | -0.005 | 0.118 |
| rs3732837AA | 0.013 | 0.022 | 0.009 | 0.58 | 0.562 | -0.03 | 0.056 |
| rs3732837T | -0.027 | 0.043 | -0.017 | -0.643 | 0.52 | -0.111 | 0.056 |
| rs9818870C | 0.097 | 0.04 | 0.063 | 2.416 | 0.016 | 0.018 | 0.176 |
| rs9818870T | 0.14 | 0.054 | 0.087 | 2.612 | 0.009 | 0.035 | 0.246 |

| **Legend:** Significant (p<0.05) |
| --- |

| ***Suppl Table 2****: Influence of gender on the relationship of CAD with MRAS gene variants* | | | | | | |  |  |
| --- | --- | --- | --- | --- | --- | --- | --- | --- |
| Parameter | B | Std. Error | t | Sig. | 95% Confidence Interval | |  |  |
|  |  |  |  |  | Lower Bound | Upper Bound |  |  |
| Intercept | .486 | .177 | 2.755 | .006 | .140 | .832 |  |  |
| [Sex=2] * rs166195C | -.275 | .103 | -2.663 | .008 | -.477 | -.073 |  |  |
| [Sex=1] * rs166195T | .177 | .076 | 2.341 | .019 | .029 | .326 |  |  |
| [Sex=2] * rs166195T | -.339 | .100 | -3.388 | .001 | -.536 | -.143 |  |  |
| [Sex=1] * rs1720819T | -.214 | .077 | -2.779 | .005 | -.365 | -.063 |  |  |
| [Sex=1] * rs1720819G | -.200 | .082 | -2.446 | .014 | -.360 | -.040 |  |  |
| [Sex=1] * rs6782181AA | .144 | .048 | 3.007 | .003 | .050 | .239 |  |  |
| [Sex=1] * rs6782181T | .143 | .049 | 2.910 | .004 | .047 | .240 |  |  |
| [Sex=2] * rs3732837AA | .124 | .048 | 2.577 | .010 | .030 | .218 |  |  |
| [Sex=1] * rs9818870C | .144 | .064 | 2.240 | .025 | .018 | .270 |  |  |
| [Sex=2] * rs9818870T | .295 | .118 | 2.512 | .012 | .065 | .526 |  |  |
|  | | | | | | | | |

| ***Suppl Table 3****: Influence of age on the relationship of CAD with MRAS gene variants* | | | | | | | |
| --- | --- | --- | --- | --- | --- | --- | --- |
| Parameter | B | Std. Error | t | Sig. | Adjusted P-Value | 95% Confidence Interval | |
|  |  |  |  |  |  | Lower Bound | Upper Bound |
| Intercept | 0.936 | 0.225 | 4.158 | 0.000 | 0.000 | 0.495 | 1.377 |
| [AGE_N=1] * rs119338AA | -0.964 | 0.269 | -3.59 | 0.000 | 0.000 | -1.49 | -0.438 |
| [AGE_N=1] * rs119338C | -0.892 | 0.273 | -3.262 | 0.001 | 0.004 | -1.428 | -0.356 |
| [AGE_N=4] * rs166195T | -0.315 | 0.152 | -2.07 | 0.038 | 0.152 | -0.614 | -0.017 |
| [AGE_N=3] * rs1720819T | -0.454 | 0.132 | -3.45 | 0.001 | 0.004 | -0.712 | -0.196 |
| [AGE_N=3] * rs1720819G | -0.382 | 0.139 | -2.745 | 0.006 | 0.024 | -0.654 | -0.109 |
| [AGE_N=3] * rs6782181AA | 0.186 | 0.078 | 2.389 | 0.017 | 0.068 | 0.033 | 0.339 |
| [AGE_N=1] * rs6782181T | 0.137 | 0.066 | 2.069 | 0.039 | 0.156 | 0.007 | 0.267 |
| [AGE_N=3] * rs6782181T | 0.185 | 0.08 | 2.3 | 0.021 | 0.084 | 0.027 | 0.343 |
| [AGE_N=2] * rs9818870C | 0.268 | 0.117 | 2.3 | 0.021 | 0.084 | 0.04 | 0.497 |
| [AGE_N=4] * rs9818870T | 0.247 | 0.124 | 1.983 | 0.047 | 0.188 | 0.003 | 0.491 |
|  | | | | | | | |

**LEGEND (AGE_N):**

**Color Legends:**

Significant after Bonferroni Adjustment

Significant before Bonferroni Adjustment

1. AGE<=46
2. 46<AGE<=57
3. 57<AGE<=66
4. AGE>66

| ***Suppl Table 4****: Influence of type 2 diabetes mellitus on the relationship of CAD with MRAS gene variants* | | | | | | |
| --- | --- | --- | --- | --- | --- | --- |
| Parameter | B | Std. Error | t | Sig. | 95% Confidence Interval | |
|  |  |  |  |  | Lower Bound | Upper Bound |
| Intercept | 0.655 | 0.131 | 5.016 | .000 | 0.399 | 0.911 |
| [DM=1] * rs166195T | -0.16 | 0.08 | -1.999 | 0.046 | -0.317 | -0.003 |
| [DM=1] * rs6782181AA | 0.116 | 0.057 | 2.044 | 0.041 | 0.005 | 0.227 |
| [DM=1] * rs9818870C | 0.185 | 0.071 | 2.624 | 0.009 | 0.047 | 0.324 |
| [DM=1] * rs9818870T | 0.271 | 0.096 | 2.821 | 0.005 | 0.083 | 0.459 |
|  | | | | | | |

| ***Suppl Table 5****: Influence of myocardial infarction on the relationship of CAD with MRAS gene variants* | | | | | | |
| --- | --- | --- | --- | --- | --- | --- |
| Parameter | B | Std. Error | t | Sig. | 95% Confidence Interval | |
|  |  |  |  |  | Lower Bound | Upper Bound |
| [MI=1] * rs1720819T | -0.261 | 0.066 | -3.975 | .000 | -0.39 | -0.132 |
| [MI=1] * rs1720819G | -0.255 | 0.069 | -3.678 | .000 | -0.391 | -0.119 |
| [MI=1] * rs6782181AA | 0.097 | 0.041 | 2.361 | 0.018 | 0.016 | 0.177 |
| [MI=1] * rs6782181T | 0.099 | 0.042 | 2.356 | 0.019 | 0.017 | 0.181 |
| [MI=1] * rs3732837AA | -0.058 | 0.029 | -2.023 | 0.043 | -0.114 | -0.002 |
| [MI=1] * rs3732837T | -0.111 | 0.053 | -2.081 | 0.037 | -0.215 | -0.006 |
| [MI=1] * rs9818870C | 0.145 | 0.055 | 2.629 | 0.009 | 0.037 | 0.252 |
| [MI=1] * rs9818870T | 0.233 | 0.072 | 3.254 | 0.001 | 0.093 | 0.374 |
|  | | | | | | |

| ***Suppl Table 6****: Influence of hypertension on the relationship of CAD with MRAS gene variants* | | | | | | |
| --- | --- | --- | --- | --- | --- | --- |
| Parameter | B | Std. Error | t | Sig. | 95% Confidence Interval | |
|  |  |  |  |  | Lower Bound | Upper Bound |
| Intercept | 0.462 | 0.11 | 4.185 | .000 | 0.246 | 0.679 |
| [HTN=1] * rs253662C | -0.069 | 0.028 | -2.452 | 0.014 | -0.125 | -0.014 |
| [HTN=1] * rs1720819T | -0.19 | 0.08 | -2.355 | 0.019 | -0.347 | -0.032 |
| [HTN=1] * rs9818870C | 0.166 | 0.059 | 2.806 | 0.005 | 0.05 | 0.283 |
| [HTN=1] * rs9818870T | 0.249 | 0.079 | 3.138 | 0.002 | 0.093 | 0.405 |
|  | | | | | | |

| ***Suppl Table 7****: Influence of obesity on the relationship of CAD with MRAS gene variants* | | | | | | |
| --- | --- | --- | --- | --- | --- | --- |
| Parameter | B | Std. Error | t | Sig. | 95% Confidence Interval | |
|  |  |  |  |  | Lower Bound | Upper Bound |
| Intercept | 0.523 | 0.142 | 3.671 | .000 | 0.244 | 0.802 |
| [OBS=1] * rs166195T | -0.251 | 0.092 | -2.72 | 0.007 | -0.433 | -0.07 |
| [OBS=1] * rs6782181AA | 0.113 | 0.057 | 1.98 | 0.048 | 0.001 | 0.225 |
| [OBS=1] * rs9818870C | 0.203 | 0.088 | 2.304 | 0.021 | 0.03 | 0.377 |
| [OBS=1] * rs9818870T | 0.346 | 0.118 | 2.941 | 0.003 | 0.116 | 0.577 |
|  | | | | | | |

***B. Analysis of MRAS gene with Obesity***

| ***Suppl Table 8****: Association of obesity with rs6782181A in presence of other risk traits* | | | | | | |
| --- | --- | --- | --- | --- | --- | --- |
| Parameter | B | Std. Error | t | Sig. | 95% Confidence Interval | |
|  |  |  |  |  | Lower Bound | Upper Bound |
| Intercept | .595 | .022 | 27.561 | .000 | .553 | .638 |
| MI | .002 | .015 | .145 | .885 | -.027 | .032 |
| CAD | .019 | .014 | 1.351 | .177 | -.009 | .047 |
| OBS | -.025 | .011 | -2.266 | .023 | -.046 | -.003 |
| Sex | .007 | .012 | .585 | .559 | -.016 | .030 |
| DM | -.015 | .011 | -1.343 | .179 | -.038 | .007 |
| AGE_N | .001 | .005 | .263 | .793 | -.009 | .011 |
| The SNP is only significant with Obesity in the presence of each trait | | | | | | |

| ***Suppl Table 9****: Relationship of obesity with rs166195Tin presence of cardiovascular risk traits* | | | | | | |
| --- | --- | --- | --- | --- | --- | --- |
| Parameter | B | Std. Error | t | Sig. | 95% Confidence Interval | |
|  |  |  |  |  | Lower Bound | Upper Bound |
| Intercept | .757 | .018 | 41.845 | .000 | .721 | .792 |
| Sex | .003 | .010 | .348 | .728 | -.016 | .023 |
| AGE_N | -.005 | .004 | -1.072 | .284 | -.013 | .004 |
| DM | .000 | .010 | -.050 | .960 | -.020 | .019 |
| OBS | -.003 | .010 | -.354 | .724 | -.022 | .016 |
|  | | | | | | |

| ***Suppl Table 10****: Relationship of obesity with rs1720819T in presence of cardiovascular risk traits* | | | | | | |
| --- | --- | --- | --- | --- | --- | --- |
| Parameter | B | Std. Error | t | Sig. | 95% Confidence Interval | |
|  |  |  |  |  | Lower Bound | Upper Bound |
| Intercept | .873 | .014 | 60.538 | .000 | .844 | .901 |
| Sex | -.004 | .008 | -.522 | .602 | -.019 | .011 |
| AGE_N | .007 | .003 | 1.922 | .055 | .000 | .013 |
| CAD | -.006 | .009 | -.717 | .473 | -.024 | .011 |
| MI | -.005 | .010 | -.510 | .610 | -.024 | .014 |
| HTN | -.002 | .009 | -.218 | .828 | -.019 | .015 |
|  | | | | | | |

| ***Suppl Table 11****: Relationship of obesity with rs1720819G in presence of cardiovascular risk traits* | | | | | | |
| --- | --- | --- | --- | --- | --- | --- |
| Parameter | B | Std. Error | t | Sig. | 95% Confidence Interval | |
|  |  |  |  |  | Lower Bound | Upper Bound |
| Intercept | .114 | .014 | 8.466 | .000 | .088 | .141 |
| Sex | .003 | .007 | .417 | .677 | -.011 | .017 |
| AGE_N | -.004 | .003 | -1.205 | .228 | -.010 | .002 |
| CAD | .003 | .009 | .306 | .759 | -.014 | .019 |
| MI | .004 | .009 | .397 | .691 | -.014 | .021 |
|  | | | | | | |

| ***Suppl Table 12****: Relationship of obesity with rs9818870C in presence of cardiovascular risk traits* | | | | | | | |
| --- | --- | --- | --- | --- | --- | --- | --- |
| Parameter | B | Std. Error | t | Sig. | 95% Confidence Interval | | |
|  |  |  |  |  | Lower Bound | Upper Bound | |
| Intercept | .878 | .015 | 58.909 | .000 | .849 | .907 | |
| Sex | .001 | .008 | .119 | .906 | -.015 | .016 | |
| AGE_N | -.001 | .004 | -.420 | .674 | -.008 | .005 | |
| CAD | .006 | .010 | .626 | .531 | -.013 | .025 | |
| MI | -.005 | .010 | -.507 | .612 | -.025 | .015 | |
| DM | -.002 | .008 | -.216 | .829 | -.017 | .014 | |
| HTN | .002 | .009 | .185 | .853 | -.016 | .019 | |
| OBS | -.001 | .007 | -.141 | .888 | -.016 | .014 | |
|  | | | | | | |  |

| ***Suppl Table 13:*** *Relationship of obesity with rs9818870T in presence of cardiovascular risk traits* | | | | | | |  |
| --- | --- | --- | --- | --- | --- | --- | --- |
| Parameter | B | Std. Error | t | Sig. | 95% Confidence Interval | |  |
|  |  |  |  |  | Lower Bound | Upper Bound |  |
| Intercept | .100 | .014 | 7.184 | .000 | .073 | .128 |  |
| Sex | -.001 | .007 | -.124 | .901 | -.015 | .014 |  |
| AGE_N | .003 | .003 | .840 | .401 | -.004 | .009 |  |
| CAD | .004 | .009 | .480 | .631 | -.013 | .022 |  |
| MI | -.001 | .009 | -.069 | .945 | -.019 | .018 |  |
| DM | .002 | .007 | .215 | .830 | -.013 | .016 |  |
| HTN | -.004 | .008 | -.485 | .627 | -.021 | .013 |  |
| OBS | .004 | .007 | .616 | .538 | -.009 | .018 |  |
|  | | | | | | | |

| ***Suppl Table 14:*** *Relationship of obesity with rs6782181T in presence of cardiovascular risk traits* | | | | | | |
| --- | --- | --- | --- | --- | --- | --- |
| Parameter | B | Std. Error | t | Sig. | 95% Confidence Interval | |
|  |  |  |  |  | Lower Bound | Upper Bound |
| Intercept | .365 | .021 | 17.773 | .000 | .324 | .405 |
| Sex | .002 | .011 | .226 | .821 | -.019 | .024 |
| AGE_N | .003 | .005 | .661 | .509 | -.006 | .013 |
| MI | -.008 | .012 | -.674 | .500 | -.030 | .015 |
|  | | | | | | |

**C. Univariate and multivariate analyses for the MRAS variants displaying significant association with the disease traits**

**MRAS versus hypercholesterolaemia**

**rs253662R (recessive mode) versus hypercholesterolaemia)**

| **rs253662R * hChol Crosstabulation** | | | | | |
| --- | --- | --- | --- | --- | --- |
|  | | | hChol | | Total |
|  |  |  | 0 | 1 |  |
| rs253662R | 1 | Count | 2556 | 1603 | 4159 |
|  |  | % within hChol | 94.9% | 96.2% | 95.4% |
|  | 2 | Count | 138 | 63 | 201 |
|  |  | % within hChol | 5.1% | 3.8% | 4.6% |
| Total | | Count | 2694 | 1666 | 4360 |
|  |  | % within hChol | 100.0% | 100.0% | 100.0% |

| **Chi-Square Tests** | | | | | |
| --- | --- | --- | --- | --- | --- |
|  | Value | df | Asymp. Sig. (2-sided) | Exact Sig. (2-sided) | Exact Sig. (1-sided) |
| Pearson Chi-Square | 4.209^a^ | 1 | .040 |  |  |
| Continuity Correction^b^ | 3.910 | 1 | .048 |  |  |
| Likelihood Ratio | 4.319 | 1 | .038 |  |  |
| Fisher's Exact Test |  |  |  | .045 | .023 |
| Linear-by-Linear Association | 4.208 | 1 | .040 |  |  |
| N of Valid Cases | 4360 |  |  |  |  |
| a. 0 cells (0.0%) have expected count less than 5. The minimum expected count is 76.80. | | | | | |
| b. Computed only for a 2x2 table | | | | | |

| **Variables in the Equation** | | | | | | | | | |
| --- | --- | --- | --- | --- | --- | --- | --- | --- | --- |
|  | | B | S.E. | Wald | df | Sig. | Exp(B) | 95% C.I.for EXP(B) | |
|  |  |  |  |  |  |  |  | Lower | Upper |
| Step 1^a^ | rs253662R(1) | -.318 | .155 | 4.178 | 1 | .041 | .728 | .537 | .987 |
|  | Constant | -.467 | .032 | 214.453 | 1 | .000 | .627 |  |  |
| a. Variable(s) entered on step 1: rs253662R. | | | | | | | | | |

| **Variables not in the Equation** | | | | | |
| --- | --- | --- | --- | --- | --- |
|  | | | Score | df | Sig. |
| Step 0 | Variables | Age | 15.976 | 1 | .000 |
|  |  | Sex(1) | 178.067 | 1 | .000 |
|  |  | CAD(1) | 143.075 | 1 | .000 |
|  |  | rs253662R(1) | .378 | 1 | .539 |
|  | Overall Statistics | | 258.912 | 4 | .000 |

| **Variables in the Equation** | | | | | | | | | |
| --- | --- | --- | --- | --- | --- | --- | --- | --- | --- |
|  | | B | S.E. | Wald | df | Sig. | Exp(B) | 95% C.I.for EXP(B) | |
|  |  |  |  |  |  |  |  | Lower | Upper |
| Step 1^a^ | Age | .000 | .002 | .001 | 1 | .971 | 1.000 | .996 | 1.005 |
|  | Sex(1) | .763 | .070 | 117.849 | 1 | .000 | 2.145 | 1.869 | 2.462 |
|  | CAD(1) | -.595 | .070 | 73.173 | 1 | .000 | .552 | .481 | .632 |
|  | rs253662R(1) | .128 | .151 | .725 | 1 | .395 | 1.137 | .846 | 1.529 |
|  | Constant | -.477 | .157 | 9.206 | 1 | .002 | .621 |  |  |
| a. Variable(s) entered on step 1: Age, Sex, CAD, rs253662R. | | | | | | | | | |

**rs253662D (dominant mode) versus hChol Crosstabulation**

| **rs253662D * hChol Crosstabulation** | | | | | |
| --- | --- | --- | --- | --- | --- |
|  | | | hChol | | Total |
|  |  |  | 0 | 1 |  |
| rs253662D | 1 | Count | 1792 | 1136 | 2928 |
|  |  | % within hChol | 66.5% | 68.2% | 67.2% |
|  | 2 | Count | 902 | 530 | 1432 |
|  |  | % within hChol | 33.5% | 31.8% | 32.8% |
| Total | | Count | 2694 | 1666 | 4360 |
|  |  | % within hChol | 100.0% | 100.0% | 100.0% |

| **Variables in the Equation** | | | | | | | | | |
| --- | --- | --- | --- | --- | --- | --- | --- | --- | --- |
|  | | B | S.E. | Wald | df | Sig. | Exp(B) | 95% C.I.for EXP(B) | |
|  |  |  |  |  |  |  |  | Lower | Upper |
| Step 1^a^ | rs253662D(1) | -.076 | .067 | 1.300 | 1 | .254 | .927 | .813 | 1.056 |
|  | Constant | -.456 | .038 | 144.454 | 1 | .000 | .634 |  |  |
| a. Variable(s) entered on step 1: rs253662D. | | | | | | | | | |

| **Variables not in the Equation** | | | | | |
| --- | --- | --- | --- | --- | --- |
|  | | | Score | df | Sig. |
| Step 0 | Variables | Age | 15.976 | 1 | .000 |
|  |  | Sex(1) | 178.067 | 1 | .000 |
|  |  | CAD(1) | 143.075 | 1 | .000 |
|  |  | rs253662D(1) | 3.434 | 1 | .064 |
|  | Overall Statistics | | 262.672 | 4 | .000 |

| **Variables in the Equation** | | | | | | | | | |
| --- | --- | --- | --- | --- | --- | --- | --- | --- | --- |
|  | | B | S.E. | Wald | df | Sig. | Exp(B) | 95% C.I.for EXP(B) | |
|  |  |  |  |  |  |  |  | Lower | Upper |
| Step 1^a^ | Age | .000 | .002 | .000 | 1 | .983 | 1.000 | .995 | 1.005 |
|  | Sex(1) | .763 | .070 | 117.736 | 1 | .000 | 2.145 | 1.869 | 2.462 |
|  | CAD(1) | -.598 | .070 | 73.879 | 1 | .000 | .550 | .480 | .630 |
|  | rs253662D(1) | .146 | .068 | 4.690 | 1 | .030 | 1.158 | 1.014 | 1.322 |
|  | Constant | -.516 | .158 | 10.591 | 1 | .001 | .597 |  |  |
| a. Variable(s) entered on step 1: Age, Sex, CAD, rs253662D. | | | | | | | | | |

**MRAS versus low HDL-Cholesterol levels**

**rs166195 allele versus low HDL levels**

| *** lHDL Crosstabulation** | | | | | |
| --- | --- | --- | --- | --- | --- |
|  | | | lHDL | | Total |
|  |  |  | 0 | 1 |  |
|  | 1 | Count | 1528 | 1174 | 2702 |
|  |  | % within lHDL | 64.4% | 61.5% | 63.1% |
|  | 2 | Count | 843 | 735 | 1578 |
|  |  | % within lHDL | 35.6% | 38.5% | 36.9% |
| Total | | Count | 2371 | 1909 | 4280 |
|  |  | % within lHDL | 100.0% | 100.0% | 100.0% |

| **Chi-Square Tests** | | | | | |
| --- | --- | --- | --- | --- | --- |
|  | Value | df | Asymp. Sig. (2-sided) | Exact Sig. (2-sided) | Exact Sig. (1-sided) |
| Pearson Chi-Square | 3.947^a^ | 1 | .047 |  |  |
| Continuity Correction^b^ | 3.821 | 1 | .051 |  |  |
| Likelihood Ratio | 3.943 | 1 | .047 |  |  |
| Fisher's Exact Test |  |  |  | .048 | .025 |
| Linear-by-Linear Association | 3.946 | 1 | .047 |  |  |
| N of Valid Cases | 4280 |  |  |  |  |
| a. 0 cells (0.0%) have expected count less than 5. The minimum expected count is 703.83. | | | | | |
| b. Computed only for a 2x2 table | | | | | |

| **Variables in the Equation** | | | | | | | | | |
| --- | --- | --- | --- | --- | --- | --- | --- | --- | --- |
|  | | B | S.E. | Wald | df | Sig. | Exp(B) | 95% C.I.for EXP(B) | |
|  |  |  |  |  |  |  |  | Lower | Upper |
| Step 1^a^ | rs166195D(1) | .126 | .064 | 3.945 | 1 | .047 | 1.135 | 1.002 | 1.286 |
|  | Constant | -.264 | .039 | 46.111 | 1 | .000 | .768 |  |  |

| a. Variable(s) entered on step 1: rs166195D. | | | | | | |
| --- | --- | --- | --- | --- | --- | --- |
| **Variables not in the Equation** | | | | | |  |
|  | | | Score | df | Sig. |  |
| Step 0 | Variables | Sex | 2.142 | 1 | .143 |  |
|  |  | Age | 3.938 | 1 | .047 |  |
|  |  | CAD(1) | 6.908 | 1 | .009 |  |
|  |  | rs166195A(1) | 1.151 | 1 | .283 |  |
|  | Overall Statistics | | 14.065 | 4 | .007 |  |

| **Variables in the Equation** | | | | | | | | | |
| --- | --- | --- | --- | --- | --- | --- | --- | --- | --- |
|  | | B | S.E. | Wald | df | Sig. | Exp(B) | 95% C.I.for EXP(B) | |
|  |  |  |  |  |  |  |  | Lower | Upper |
| Step 1^a^ | Sex | .144 | .066 | 4.724 | 1 | .030 | 1.155 | 1.014 | 1.315 |
|  | Age | .003 | .002 | 1.270 | 1 | .260 | 1.003 | .998 | 1.007 |
|  | CAD(1) | -.171 | .068 | 6.366 | 1 | .012 | .843 | .738 | .963 |
|  | rs166195A(1) | -.076 | .073 | 1.103 | 1 | .294 | .926 | .803 | 1.068 |
|  | Constant | -2.003 | .164 | 149.404 | 1 | .000 | .135 |  |  |
| a. Variable(s) entered on step 1: Sex, Age, CAD, rs166195A. | | | | | | | | | |

| **Variables in the Equation** | | | | | | | | | |
| --- | --- | --- | --- | --- | --- | --- | --- | --- | --- |
|  | | B | S.E. | Wald | df | Sig. | Exp(B) | 95% C.I.for EXP(B) | |
|  |  |  |  |  |  |  |  | Lower | Upper |
| Step 1^a^ | Sex | .190 | .070 | 7.386 | 1 | .007 | 1.210 | 1.054 | 1.387 |
|  | Age | .013 | .002 | 30.013 | 1 | .000 | 1.013 | 1.008 | 1.018 |
|  | CAD(1) | -.761 | .070 | 116.721 | 1 | .000 | .467 | .407 | .536 |
|  | rs166195D(1) | -.011 | .066 | .028 | 1 | .867 | .989 | .869 | 1.126 |
|  | Constant | -1.137 | .174 | 42.876 | 1 | .000 | .321 |  |  |
| a. Variable(s) entered on step 1: Sex, Age, CAD, rs166195D. | | | | | | | | | |

**rs253662D versus*low HDL-choleterol**

| **rs253662D * lHDL Crosstabulation** | | | | | |
| --- | --- | --- | --- | --- | --- |
|  | | | lHDL | | Total |
|  |  |  | 0 | 1 |  |
| rs253662D | 1 | Count | 1614 | 1247 | 2861 |
|  |  | % within lHDL | 68.2% | 65.6% | 67.0% |
|  | 2 | Count | 751 | 655 | 1406 |
|  |  | % within lHDL | 31.8% | 34.4% | 33.0% |
| Total | | Count | 2365 | 1902 | 4267 |
|  |  | % within lHDL | 100.0% | 100.0% | 100.0% |

| **Chi-Square Tests** | | | | | |
| --- | --- | --- | --- | --- | --- |
|  | Value | df | Asymp. Sig. (2-sided) | Exact Sig. (2-sided) | Exact Sig. (1-sided) |
| Pearson Chi-Square | 3.434^a^ | 1 | .064 |  |  |
| Continuity Correction^b^ | 3.314 | 1 | .069 |  |  |
| Likelihood Ratio | 3.430 | 1 | .064 |  |  |
| Fisher's Exact Test |  |  |  | .067 | .034 |
| Linear-by-Linear Association | 3.433 | 1 | .064 |  |  |
| N of Valid Cases | 4267 |  |  |  |  |
| a. 0 cells (0.0%) have expected count less than 5. The minimum expected count is 626.72. | | | | | |
| b. Computed only for a 2x2 table | | | | | |

| **Variables in the Equation** | | | | | | | | | |
| --- | --- | --- | --- | --- | --- | --- | --- | --- | --- |
|  | | B | S.E. | Wald | df | Sig. | Exp(B) | 95% C.I.for EXP(B) | |
|  |  |  |  |  |  |  |  | Lower | Upper |
| Step 1^a^ | rs253662D(1) | .121 | .065 | 3.433 | 1 | .064 | 1.129 | .993 | 1.283 |
|  | Constant | -.258 | .038 | 46.817 | 1 | .000 | .773 |  |  |
| a. Variable(s) entered on step 1: rs253662D. | | | | | | | | | |

| **Variables not in the Equation** | | | | | |
| --- | --- | --- | --- | --- | --- |
|  | | | Score | df | Sig. |
| Step 0 | Variables | Sex | .284 | 1 | .594 |
|  |  | Age | 89.732 | 1 | .000 |
|  |  | CAD(1) | 174.742 | 1 | .000 |
|  |  | rs253662R(1) | 4.209 | 1 | .040 |
|  | Overall Statistics | | 213.191 | 4 | .000 |

| **Variables in the Equation** | | | | | | | | | |
| --- | --- | --- | --- | --- | --- | --- | --- | --- | --- |
|  | | B | S.E. | Wald | df | Sig. | Exp(B) | 95% C.I.for EXP(B) | |
|  |  |  |  |  |  |  |  | Lower | Upper |
| Step 1^a^ | Sex | .193 | .070 | 7.612 | 1 | .006 | 1.213 | 1.057 | 1.391 |
|  | Age | .013 | .002 | 29.266 | 1 | .000 | 1.013 | 1.008 | 1.018 |
|  | CAD(1) | -.772 | .071 | 119.800 | 1 | .000 | .462 | .402 | .530 |
|  | rs253662R(1) | -.307 | .159 | 3.714 | 1 | .054 | .736 | .539 | 1.005 |
|  | Constant | -1.117 | .172 | 41.945 | 1 | .000 | .327 |  |  |
| a. Variable(s) entered on step 1: Sex, Age, CAD, rs253662R. | | | | | | | | | |

**MRAS versus high LDL levels**

**rs253662 Allele versus* high LDL-cholesterol Crosstabulation**

| **rs253662A * hLDL Crosstabulation** | | | | | |
| --- | --- | --- | --- | --- | --- |
|  | | | hLDL | | Total |
|  |  |  | 0 | 1 |  |
| rs253662A | 1 | Count | 5874 | 1047 | 6921 |
|  |  | % within hLDL | 80.8% | 83.8% | 81.2% |
|  | 2 | Count | 1398 | 203 | 1601 |
|  |  | % within hLDL | 19.2% | 16.2% | 18.8% |
| Total | | Count | 7272 | 1250 | 8522 |
|  |  | % within hLDL | 100.0% | 100.0% | 100.0% |

| **Chi-Square Tests** | | | | | |
| --- | --- | --- | --- | --- | --- |
|  | Value | df | Asymp. Sig. (2-sided) | Exact Sig. (2-sided) | Exact Sig. (1-sided) |
| Pearson Chi-Square | 6.227^a^ | 1 | .013 |  |  |
| Continuity Correction^b^ | 6.033 | 1 | .014 |  |  |
| Likelihood Ratio | 6.421 | 1 | .011 |  |  |
| Fisher's Exact Test |  |  |  | .012 | .006 |
| Linear-by-Linear Association | 6.226 | 1 | .013 |  |  |
| N of Valid Cases | 8522 |  |  |  |  |
| a. 0 cells (0.0%) have expected count less than 5. The minimum expected count is 234.83. | | | | | |
| b. Computed only for a 2x2 table | | | | | |

| **Variables in the Equation** | | | | | | | | | |
| --- | --- | --- | --- | --- | --- | --- | --- | --- | --- |
|  | | B | S.E. | Wald | df | Sig. | Exp(B) | 95% C.I.for EXP(B) | |
|  |  |  |  |  |  |  |  | Lower | Upper |
| Step 1^a^ | rs253662A(1) | -.205 | .082 | 6.210 | 1 | .013 | .815 | .693 | .957 |
|  | Constant | -1.725 | .034 | 2642.969 | 1 | .000 | .178 |  |  |
| a. Variable(s) entered on step 1: rs253662A. | | | | | | | | | |

| **Variables not in the Equation** | | | | | |
| --- | --- | --- | --- | --- | --- |
|  | | | Score | df | Sig. |
| Step 0 | Variables | Sex | 1.314 | 1 | .252 |
|  |  | Age | 3.560 | 1 | .059 |
|  |  | CAD(1) | 5.735 | 1 | .017 |
|  |  | rs253662A(1) | 6.227 | 1 | .013 |
|  | Overall Statistics | | 16.182 | 4 | .003 |

| **Variables in the Equation** | | | | | | | | | |
| --- | --- | --- | --- | --- | --- | --- | --- | --- | --- |
|  | | B | S.E. | Wald | df | Sig. | Exp(B) | 95% C.I.for EXP(B) | |
|  |  |  |  |  |  |  |  | Lower | Upper |
| Step 1^a^ | Sex | .118 | .066 | 3.156 | 1 | .076 | 1.125 | .988 | 1.281 |
|  | Age | .003 | .002 | 1.246 | 1 | .264 | 1.003 | .998 | 1.007 |
|  | CAD(1) | -.148 | .068 | 4.764 | 1 | .029 | .863 | .755 | .985 |
|  | rs253662A(1) | -.202 | .082 | 6.023 | 1 | .014 | .817 | .695 | .960 |
|  | Constant | -1.958 | .164 | 142.999 | 1 | .000 | .141 |  |  |
| a. Variable(s) entered on step 1: Sex, Age, CAD, rs253662A. | | | | | | | | | |

**rs253662 Dominant mode versus high LDL-cholesterol**

| **rs253662D * hLDL Crosstabulation** | | | | | |
| --- | --- | --- | --- | --- | --- |
|  | | | hLDL | | Total |
|  |  |  | 0 | 1 |  |
| rs253662D | 1 | Count | 2414 | 443 | 2857 |
|  |  | % within hLDL | 66.4% | 70.9% | 67.0% |
|  | 2 | Count | 1222 | 182 | 1404 |
|  |  | % within hLDL | 33.6% | 29.1% | 33.0% |
| Total | | Count | 3636 | 625 | 4261 |
|  |  | % within hLDL | 100.0% | 100.0% | 100.0% |

| **Chi-Square Tests** | | | | | |
| --- | --- | --- | --- | --- | --- |
|  | Value | df | Asymp. Sig. (2-sided) | Exact Sig. (2-sided) | Exact Sig. (1-sided) |
| Pearson Chi-Square | 4.863^a^ | 1 | .027 |  |  |
| Continuity Correction^b^ | 4.662 | 1 | .031 |  |  |
| Likelihood Ratio | 4.950 | 1 | .026 |  |  |
| Fisher's Exact Test |  |  |  | .027 | .015 |
| Linear-by-Linear Association | 4.862 | 1 | .027 |  |  |
| N of Valid Cases | 4261 |  |  |  |  |
| a. 0 cells (0.0%) have expected count less than 5. The minimum expected count is 205.94. | | | | | |
| b. Computed only for a 2x2 table | | | | | |

| **Variables in the Equation** | | | | | | | | | |
| --- | --- | --- | --- | --- | --- | --- | --- | --- | --- |
|  | | B | S.E. | Wald | df | Sig. | Exp(B) | 95% C.I.for EXP(B) | |
|  |  |  |  |  |  |  |  | Lower | Upper |
| Step 1^a^ | rs253662D(1) | -.209 | .095 | 4.851 | 1 | .028 | .812 | .674 | .977 |
|  | Constant | -1.695 | .052 | 1075.998 | 1 | .000 | .184 |  |  |
| a. Variable(s) entered on step 1: rs253662D. | | | | | | | | | |

| **Variables not in the Equation** | | | | | |
| --- | --- | --- | --- | --- | --- |
|  | | | Score | df | Sig. |
| Step 0 | Variables | Sex | .657 | 1 | .418 |
|  |  | Age | 1.780 | 1 | .182 |
|  |  | CAD(1) | 2.868 | 1 | .090 |
|  |  | rs253662D(1) | 4.863 | 1 | .027 |
|  | Overall Statistics | | 9.777 | 4 | .044 |

| **Variables in the Equation** | | | | | | | | | |
| --- | --- | --- | --- | --- | --- | --- | --- | --- | --- |
|  | | B | S.E. | Wald | df | Sig. | Exp(B) | 95% C.I.for EXP(B) | |
|  |  |  |  |  |  |  |  | Lower | Upper |
| Step 1^a^ | Sex | .117 | .094 | 1.561 | 1 | .211 | 1.124 | .936 | 1.351 |
|  | Age | .003 | .003 | .638 | 1 | .425 | 1.003 | .996 | 1.009 |
|  | CAD(1) | -.146 | .096 | 2.316 | 1 | .128 | .864 | .716 | 1.043 |
|  | rs253662D(1) | -.206 | .095 | 4.694 | 1 | .030 | .814 | .676 | .981 |
|  | Constant | -1.931 | .232 | 69.012 | 1 | .000 | .145 |  |  |
| a. Variable(s) entered on step 1: Sex, Age, CAD, rs253662D. | | | | | | | | | |

| **rs6782181AG * hLDL Crosstabulation** | | | | | |
| --- | --- | --- | --- | --- | --- |
|  | | | hLDL | | Total |
|  |  |  | 0 | 1 |  |
| rs6782181AG | 1 | Count | 4426 | 791 | 5217 |
|  |  | % within hLDL | 61.3% | 64.4% | 61.8% |
|  | 2 | Count | 2794 | 437 | 3231 |
|  |  | % within hLDL | 38.7% | 35.6% | 38.2% |
| Total | | Count | 7220 | 1228 | 8448 |
|  |  | % within hLDL | 100.0% | 100.0% | 100.0% |

| **Chi-Square Tests** | | | | | |
| --- | --- | --- | --- | --- | --- |
|  | Value | df | Asymp. Sig. (2-sided) | Exact Sig. (2-sided) | Exact Sig. (1-sided) |
| Pearson Chi-Square | 4.303^a^ | 1 | .038 |  |  |
| Continuity Correction^b^ | 4.172 | 1 | .041 |  |  |
| Likelihood Ratio | 4.337 | 1 | .037 |  |  |
| Fisher's Exact Test |  |  |  | .039 | .020 |
| Linear-by-Linear Association | 4.302 | 1 | .038 |  |  |
| N of Valid Cases | 8448 |  |  |  |  |
| a. 0 cells (0.0%) have expected count less than 5. The minimum expected count is 469.66. | | | | | |
| b. Computed only for a 2x2 table | | | | | |

**rs6782181D versus high LDL-cholesterol level**

| **rs6782181D * hLDL Crosstabulation** | | | | | |
| --- | --- | --- | --- | --- | --- |
|  | | | hLDL | | Total |
|  |  |  | 0 | 1 |  |
| rs6782181D | 1 | Count | 1395 | 268 | 1663 |
|  |  | % within hLDL | 38.6% | 43.6% | 39.4% |
|  | 2 | Count | 2215 | 346 | 2561 |
|  |  | % within hLDL | 61.4% | 56.4% | 60.6% |
| Total | | Count | 3610 | 614 | 4224 |
|  |  | % within hLDL | 100.0% | 100.0% | 100.0% |

| **Chi-Square Tests** | | | | | |
| --- | --- | --- | --- | --- | --- |
|  | Value | df | Asymp. Sig. (2-sided) | Exact Sig. (2-sided) | Exact Sig. (1-sided) |
| Pearson Chi-Square | 5.508^a^ | 1 | .019 |  |  |
| Continuity Correction^b^ | 5.300 | 1 | .021 |  |  |
| Likelihood Ratio | 5.457 | 1 | .019 |  |  |
| Fisher's Exact Test |  |  |  | .020 | .011 |
| Linear-by-Linear Association | 5.507 | 1 | .019 |  |  |
| N of Valid Cases | 4224 |  |  |  |  |
| a. 0 cells (0.0%) have expected count less than 5. The minimum expected count is 241.73. | | | | | |
| b. Computed only for a 2x2 table | | | | | |

| **Variables in the Equation** | | | | | | | | | |
| --- | --- | --- | --- | --- | --- | --- | --- | --- | --- |
|  | | B | S.E. | Wald | df | Sig. | Exp(B) | 95% C.I.for EXP(B) | |
|  |  |  |  |  |  |  |  | Lower | Upper |
| Step 1^a^ | rs6782181D(1) | -.207 | .088 | 5.496 | 1 | .019 | .813 | .684 | .967 |
|  | Constant | -1.650 | .067 | 611.797 | 1 | .000 | .192 |  |  |
| a. Variable(s) entered on step 1: rs6782181D. | | | | | | | | | |

| **Variables not in the Equation** | | | | | |
| --- | --- | --- | --- | --- | --- |
|  | | | Score | df | Sig. |
| Step 0 | Variables | Sex | .947 | 1 | .331 |
|  |  | Age | 1.430 | 1 | .232 |
|  |  | CAD(1) | 2.566 | 1 | .109 |
|  |  | rs6782181D(1) | 5.508 | 1 | .019 |
|  | Overall Statistics | | 10.466 | 4 | .033 |

| **Variables in the Equation** | | | | | | | | | |
| --- | --- | --- | --- | --- | --- | --- | --- | --- | --- |
|  | | B | S.E. | Wald | df | Sig. | Exp(B) | 95% C.I.for EXP(B) | |
|  |  |  |  |  |  |  |  | Lower | Upper |
| Step 1^a^ | Sex | .134 | .094 | 2.021 | 1 | .155 | 1.144 | .950 | 1.376 |
|  | Age | .002 | .003 | .476 | 1 | .490 | 1.002 | .996 | 1.009 |
|  | CAD(1) | -.147 | .097 | 2.326 | 1 | .127 | .863 | .714 | 1.043 |
|  | rs6782181D(1) | -.206 | .088 | 5.458 | 1 | .019 | .814 | .684 | .967 |
|  | Constant | -1.888 | .237 | 63.439 | 1 | .000 | .151 |  |  |
| a. Variable(s) entered on step 1: Sex, Age, CAD, rs6782181D. | | | | | | | | | |

| **Variables not in the Equation** | | | | | |
| --- | --- | --- | --- | --- | --- |
|  | | | Score | df | Sig. |
| Step 0 | Variables | Age | 2.860 | 1 | .091 |
|  |  | Sex(1) | 1.893 | 1 | .169 |
|  |  | CAD(1) | 5.133 | 1 | .023 |
|  |  | rs6782181A(1) | 4.303 | 1 | .038 |
|  | Overall Statistics | | 14.225 | 4 | .007 |

| **Variables in the Equation** | | | | | | | | | |
| --- | --- | --- | --- | --- | --- | --- | --- | --- | --- |
|  | | B | S.E. | Wald | df | Sig. | Exp(B) | 95% C.I.for EXP(B) | |
|  |  |  |  |  |  |  |  | Lower | Upper |
| Step 1^a^ | Age | .002 | .002 | .926 | 1 | .336 | 1.002 | .998 | 1.007 |
|  | Sex(1) | -.133 | .067 | 3.961 | 1 | .047 | .876 | .768 | .998 |
|  | CAD(1) | -.149 | .068 | 4.744 | 1 | .029 | .862 | .754 | .985 |
|  | rs6782181A(1) | -.132 | .064 | 4.227 | 1 | .040 | .876 | .772 | .994 |
|  | Constant | -1.691 | .154 | 120.756 | 1 | .000 | .184 |  |  |
| a. Variable(s) entered on step 1: Age, Sex, CAD, rs6782181A. | | | | | | | | | |

| **rs6782181D * hLDL Crosstabulation** | | | | | |
| --- | --- | --- | --- | --- | --- |
|  | | | hLDL | | Total |
|  |  |  | 0 | 1 |  |
| rs6782181D | 1 | Count | 1395 | 268 | 1663 |
|  |  | % within hLDL | 38.6% | 43.6% | 39.4% |
|  | 2 | Count | 2215 | 346 | 2561 |
|  |  | % within hLDL | 61.4% | 56.4% | 60.6% |
| Total | | Count | 3610 | 614 | 4224 |
|  |  | % within hLDL | 100.0% | 100.0% | 100.0% |

| **Chi-Square Tests** | | | | | |
| --- | --- | --- | --- | --- | --- |
|  | Value | df | Asymp. Sig. (2-sided) | Exact Sig. (2-sided) | Exact Sig. (1-sided) |
| Pearson Chi-Square | 5.508^a^ | 1 | .019 |  |  |
| Continuity Correction^b^ | 5.300 | 1 | .021 |  |  |
| Likelihood Ratio | 5.457 | 1 | .019 |  |  |
| Fisher's Exact Test |  |  |  | .020 | .011 |
| Linear-by-Linear Association | 5.507 | 1 | .019 |  |  |
| N of Valid Cases | 4224 |  |  |  |  |
| a. 0 cells (0.0%) have expected count less than 5. The minimum expected count is 241.73. | | | | | |
| b. Computed only for a 2x2 table | | | | | |

| **Variables in the Equation** | | | | | | | | | |
| --- | --- | --- | --- | --- | --- | --- | --- | --- | --- |
|  | | B | S.E. | Wald | df | Sig. | Exp(B) | 95% C.I.for EXP(B) | |
|  |  |  |  |  |  |  |  | Lower | Upper |
| Step 1^a^ | rs6782181D(1) | -.207 | .088 | 5.496 | 1 | .019 | .813 | .684 | .967 |
|  | Constant | -1.650 | .067 | 611.797 | 1 | .000 | .192 |  |  |
| a. Variable(s) entered on step 1: rs6782181D. | | | | | | | | | |

| **Variables in the Equation** | | | | | | | | | |
| --- | --- | --- | --- | --- | --- | --- | --- | --- | --- |
|  | | B | S.E. | Wald | df | Sig. | Exp(B) | 95% C.I.for EXP(B) | |
|  |  |  |  |  |  |  |  | Lower | Upper |
| Step 1^a^ | rs6782181A(1) | -.133 | .064 | 4.298 | 1 | .038 | .875 | .772 | .993 |
|  | Constant | -1.722 | .039 | 1989.802 | 1 | .000 | .179 |  |  |
| a. Variable(s) entered on step 1: rs6782181A. | | | | | | | | | |

| **Variables not in the Equation** | | | | | |
| --- | --- | --- | --- | --- | --- |
|  | | | Score | df | Sig. |
| Step 0 | Variables | Sex | .947 | 1 | .331 |
|  |  | Age | 1.430 | 1 | .232 |
|  |  | CAD(1) | 2.566 | 1 | .109 |
|  |  | rs6782181D(1) | 5.508 | 1 | .019 |
|  | Overall Statistics | | 10.466 | 4 | .033 |

| **Variables in the Equation** | | | | | | | | | |
| --- | --- | --- | --- | --- | --- | --- | --- | --- | --- |
|  | | B | S.E. | Wald | df | Sig. | Exp(B) | 95% C.I.for EXP(B) | |
|  |  |  |  |  |  |  |  | Lower | Upper |
| Step 1^a^ | Sex | .134 | .094 | 2.021 | 1 | .155 | 1.144 | .950 | 1.376 |
|  | Age | .002 | .003 | .476 | 1 | .490 | 1.002 | .996 | 1.009 |
|  | CAD(1) | -.147 | .097 | 2.326 | 1 | .127 | .863 | .714 | 1.043 |
|  | rs6782181D(1) | -.206 | .088 | 5.458 | 1 | .019 | .814 | .684 | .967 |
|  | Constant | -1.888 | .237 | 63.439 | 1 | .000 | .151 |  |  |
| a. Variable(s) entered on step 1: Sex, Age, CAD, rs6782181D. | | | | | | | | | |

| **Variables not in the Equation** | | | | | |
| --- | --- | --- | --- | --- | --- |
|  | | | Score | df | Sig. |
| Step 0 | Variables | Age | 1.430 | 1 | .232 |
|  |  | Sex(1) | .947 | 1 | .331 |
|  |  | CAD(1) | 2.566 | 1 | .109 |
|  |  | rs6782181D(1) | 5.508 | 1 | .019 |
|  | Overall Statistics | | 10.466 | 4 | .033 |

| **Variables in the Equation** | | | | | | | | | |
| --- | --- | --- | --- | --- | --- | --- | --- | --- | --- |
|  | | B | S.E. | Wald | df | Sig. | Exp(B) | 95% C.I.for EXP(B) | |
|  |  |  |  |  |  |  |  | Lower | Upper |
| Step 1^a^ | Age | .002 | .003 | .476 | 1 | .490 | 1.002 | .996 | 1.009 |
|  | Sex(1) | -.134 | .094 | 2.021 | 1 | .155 | .874 | .727 | 1.052 |
|  | CAD(1) | -.147 | .097 | 2.326 | 1 | .127 | .863 | .714 | 1.043 |
|  | rs6782181D(1) | .206 | .088 | 5.458 | 1 | .019 | 1.229 | 1.034 | 1.462 |
|  | Constant | -1.826 | .219 | 69.499 | 1 | .000 | .161 |  |  |
| a. Variable(s) entered on step 1: Age, Sex, CAD, rs6782181D. | | | | | | | | | |

Hypertriglyceridaemia

**rs6782181Allele versus hypertriglyceridaema**

| **rs6782181AG * hTG Crosstabulation** | | | | | |
| --- | --- | --- | --- | --- | --- |
|  | | | hTG | | Total |
|  |  |  | 0 | 1 |  |
| rs6782181AG | 1 | Count | 3853 | 1369 | 5222 |
|  |  | % within hTG | 62.3% | 60.3% | 61.8% |
|  | 2 | Count | 2331 | 903 | 3234 |
|  |  | % within hTG | 37.7% | 39.7% | 38.2% |
| Total | | Count | 6184 | 2272 | 8456 |
|  |  | % within hTG | 100.0% | 100.0% | 100.0% |

| **Chi-Square Tests** | | | | | |
| --- | --- | --- | --- | --- | --- |
|  | Value | df | Asymp. Sig. (2-sided) | Exact Sig. (2-sided) | Exact Sig. (1-sided) |
| Pearson Chi-Square | 2.958^a^ | 1 | .085 |  |  |
| Continuity Correction^b^ | 2.872 | 1 | .090 |  |  |
| Likelihood Ratio | 2.950 | 1 | .086 |  |  |
| Fisher's Exact Test |  |  |  | .086 | .045 |
| Linear-by-Linear Association | 2.958 | 1 | .085 |  |  |
| N of Valid Cases | 8456 |  |  |  |  |
| a. 0 cells (0.0%) have expected count less than 5. The minimum expected count is 868.93. | | | | | |
| b. Computed only for a 2x2 table | | | | | |

| **Variables in the Equation** | | | | | | | | | |
| --- | --- | --- | --- | --- | --- | --- | --- | --- | --- |
|  | | B | S.E. | Wald | df | Sig. | Exp(B) | 95% C.I.for EXP(B) | |
|  |  |  |  |  |  |  |  | Lower | Upper |
| Step 1^a^ | rs6782181A(1) | .086 | .050 | 2.958 | 1 | .085 | 1.090 | .988 | 1.203 |
|  | Constant | -1.035 | .031 | 1081.570 | 1 | .000 | .355 |  |  |
| a. Variable(s) entered on step 1: rs6782181A. | | | | | | | | | |

| **rs6782181AG * hTG Crosstabulation** | | | | | |
| --- | --- | --- | --- | --- | --- |
|  | | | hTG | | Total |
|  |  |  | 0 | 1 |  |
| rs6782181AG | 1 | Count | 2622 | 937 | 3559 |
|  |  | % within hTG | 84.8% | 82.5% | 84.2% |
|  | 2 | Count | 470 | 199 | 669 |
|  |  | % within hTG | 15.2% | 17.5% | 15.8% |
| Total | | Count | 3092 | 1136 | 4228 |
|  |  | % within hTG | 100.0% | 100.0% | 100.0% |

| **Chi-Square Tests** | | | | | |
| --- | --- | --- | --- | --- | --- |
|  | Value | df | Asymp. Sig. (2-sided) | Exact Sig. (2-sided) | Exact Sig. (1-sided) |
| Pearson Chi-Square | 3.349^a^ | 1 | .067 |  |  |
| Continuity Correction^b^ | 3.177 | 1 | .075 |  |  |
| Likelihood Ratio | 3.291 | 1 | .070 |  |  |
| Fisher's Exact Test |  |  |  | .071 | .038 |
| Linear-by-Linear Association | 3.348 | 1 | .067 |  |  |
| N of Valid Cases | 4228 |  |  |  |  |
| a. 0 cells (0.0%) have expected count less than 5. The minimum expected count is 179.75. | | | | | |
| b. Computed only for a 2x2 table | | | | | |

| **Variables in the Equation** | | | | | | | | | |
| --- | --- | --- | --- | --- | --- | --- | --- | --- | --- |
|  | | B | S.E. | Wald | df | Sig. | Exp(B) | 95% C.I.for EXP(B) | |
|  |  |  |  |  |  |  |  | Lower | Upper |
| Step 1^a^ | rs6782181A(1) | .170 | .093 | 3.343 | 1 | .067 | 1.185 | .988 | 1.421 |
|  | Constant | -1.029 | .038 | 730.942 | 1 | .000 | .357 |  |  |
| a. Variable(s) entered on step 1: rs6782181A. | | | | | | | | | |

| **Variables not in the Equation** | | | | | |
| --- | --- | --- | --- | --- | --- |
|  | | | Score | df | Sig. |
| Step 0 | Variables | Sex(1) | 20.862 | 1 | .000 |
|  |  | Age | .921 | 1 | .337 |
|  |  | CAD(1) | 79.146 | 1 | .000 |
|  |  | rs6782181A(1) | 3.349 | 1 | .067 |
|  | Overall Statistics | | 93.994 | 4 | .000 |

| **Variables in the Equation** | | | | | | | |
| --- | --- | --- | --- | --- | --- | --- | --- |
|  | | B | S.E. | Wald | df | Sig. | Exp(B) |
| Step 1^a^ | Sex(1) | .191 | .078 | 5.938 | 1 | .015 | 1.211 |
|  | Age | -.006 | .003 | 5.312 | 1 | .021 | .994 |
|  | CAD(1) | -.658 | .079 | 68.553 | 1 | .000 | .518 |
|  | rs6782181A(1) | .190 | .094 | 4.097 | 1 | .043 | 1.209 |
|  | Constant | -.548 | .176 | 9.709 | 1 | .002 | .578 |
| a. Variable(s) entered on step 1: Sex, Age, CAD, rs6782181A. | | | | | | | |

| **Variables not in the Equation** | | | | | |
| --- | --- | --- | --- | --- | --- |
|  | | | Score | df | Sig. |
| Step 0 | Variables | Sex(1) | 20.862 | 1 | .000 |
|  |  | Age | .921 | 1 | .337 |
|  |  | CAD(1) | 79.146 | 1 | .000 |
|  |  | rs6782181A(1) | 3.349 | 1 | .067 |
|  | Overall Statistics | | 93.994 | 4 | .000 |

| **Variables in the Equation** | | | | | | | | | |
| --- | --- | --- | --- | --- | --- | --- | --- | --- | --- |
|  | | B | S.E. | Wald | df | Sig. | Exp(B) | 95% C.I.for EXP(B) | |
|  |  |  |  |  |  |  |  | Lower | Upper |
| Step 1^a^ | Sex(1) | .191 | .078 | 5.938 | 1 | .015 | 1.211 | 1.038 | 1.412 |
|  | Age | -.006 | .003 | 5.312 | 1 | .021 | .994 | .989 | .999 |
|  | CAD(1) | -.658 | .079 | 68.553 | 1 | .000 | .518 | .443 | .605 |
|  | rs6782181A(1) | .190 | .094 | 4.097 | 1 | .043 | 1.209 | 1.006 | 1.454 |
|  | Constant | -.548 | .176 | 9.709 | 1 | .002 | .578 |  |  |
| a. Variable(s) entered on step 1: Sex, Age, CAD, rs6782181A. | | | | | | | | | |
